# The most economical arthroscopic suture fixation for tibial intercondylar eminence avulsion fracture without any implant

**DOI:** 10.1186/s13018-022-03219-w

**Published:** 2022-06-25

**Authors:** Libo Yuan, Rongmao Shi, Zhian Chen, Wei Ding, Hongbo Tan

**Affiliations:** 1 Department of Orthopaedics, People’s Liberation Army Joint Logistic Support Force 920Th Hospital, No. 212 Daguan Road, Xi Shan District, Kunming, 650032 Yunnan China; 2 Kunming Medical University, Kunming, 650032 Yunnan China; 3 College of Medicine Technology, Yunnan Medical Health College, Kunming, 650106 Yunnan China

**Keywords:** Tibial intercondylar eminence, Avulsion fracture, Arthroscopic, Suture, Fixation

## Abstract

**Background:**

Avulsion fracture of the tibial intercondylar eminence is a rare injury, which mainly occurs in adolescents aged 8–14 years and in those with immature bones. The current commonly used surgery may result in severe surgical trauma, affecting knee joint function and accompanied by serious complications. In this study, we described an all-inside and all-epiphyseal arthroscopic suture fixation technique for a patient to treat tibial intercondylar eminence fracture.

**Methods:**

ETHIBOND EXCEL-coated braided polyester sutures were used for fixation. Three ETHIBOND sutures were passed through the ACL at 2, 6 and 10 o’clock of the footprint of the ACL and made a cinch-knot loop separately. Under the guidance of ACL tibial locator, three corresponding tibial tunnels were drilled with K-wires at 2, 6 and 10 o’clock of the fracture bed, and the two ends of the suture were pulled out through the tunnel with double-folded steel wire heads. After reduction of the tibial eminence, three sutures were tightened and tied to the medial aspect of the tibial tubercle.

**Results:**

After all the surgical treatments surgically performed by this method and following a standard postoperative protocol, our patient's ROM, stability, and functional structural scores all improved significantly.

**Conclusion:**

This three-point suture technique provides a suitable reduction and stable fixation and is suitable for patients with all types of avulsion fractures of the tibial intercondylar eminence.

## Background

The point of bony prominence in the central region of the tibial plateau, called the tibial eminence or tibial spine, is the site of attachment of the anterior cruciate ligament (ACL). Tibial eminence fracture is a rare injury occurring primarily in adolescence, and the mechanism of injury is knee hyperextension or strong rotation concurrent with knee flexion. For adult with mature skeleton, similar injury mechanisms often resulted in rupture of ACL [1]. Tibial eminence avulsion fractures are more common in children and adolescents between the ages of 8–14, with an incidence of 3/100,000 in children and 2% of knee injuries in children [2]. The reason for the high incidence of this injury in children may be that the tibial plateau with incomplete ossification is weak, leading to ACL footprint avulsion more easily than ACL rupture [3]. The incidence of tibial intercondylar crest fracture in adults is increasing with the increase in sports injuries and traffic accidents. These fractures can recover well in both children and adults if treated effectively at an early stage of tibial eminence avulsion injury [4, 5].

The avulsion fracture of tibial intercondylar crest can be classified according to Meyers–McKeever–Zaricnyj classification: Type I fracture means that the fragments do not fall off from their original bed; type II, partial dislocation of the fragment, but most of the avulsion fragments remained well attached; and type III fracture is displaced. Type IV fracture is displaced and comminuted [6]. Type I fractures are usually treated non-operatively with plaster immobilization, while type II fractures can be treated with closed reduction and cast immobilization at the same time [1]. Recent studies have demonstrated a similar incidence of complications for type II fractures treated by non-surgical treatment and surgical treatment, a higher incidence of residual relaxation and subsequent surgery of the tibial spine and ACL in patients treated by non-surgical treatment, and a higher incidence of joint fibrosis in patients treated by surgical treatment [7]. Therefore, surgical treatment is generally recommended for patients with type II fractures, in addition to anatomical reduction through knee extension and immobilization [6]. Surgical reduction and fixation are mostly the optimal treatment options for type III and type IV fractures [1, 8].

With the advancement of the times and the development of medical technology, arthroscopic technique is considered to be the gold standard for the treatment of tibial intercondylar ridge avulsion fractures. A variety of techniques, including metal screws [9], stainless steel wires [10], K-wires [11], suture anchors [12, 13], sutures [14], bioabsorbable nails [15], and steel wires with suture disks [16], are advocated for treating such injuries. Each approach has its own advantages and disadvantages. The current reports have shown the obvious advantages of arthroscopy in the surgical application, but the operation is costly and cannot be widely implemented [3, 17]. Therefore, it is important to explore an economical surgical approach to the treatment of tibial fractures.

## Materials and methods

### Surgical technique

**Fig. 1:**
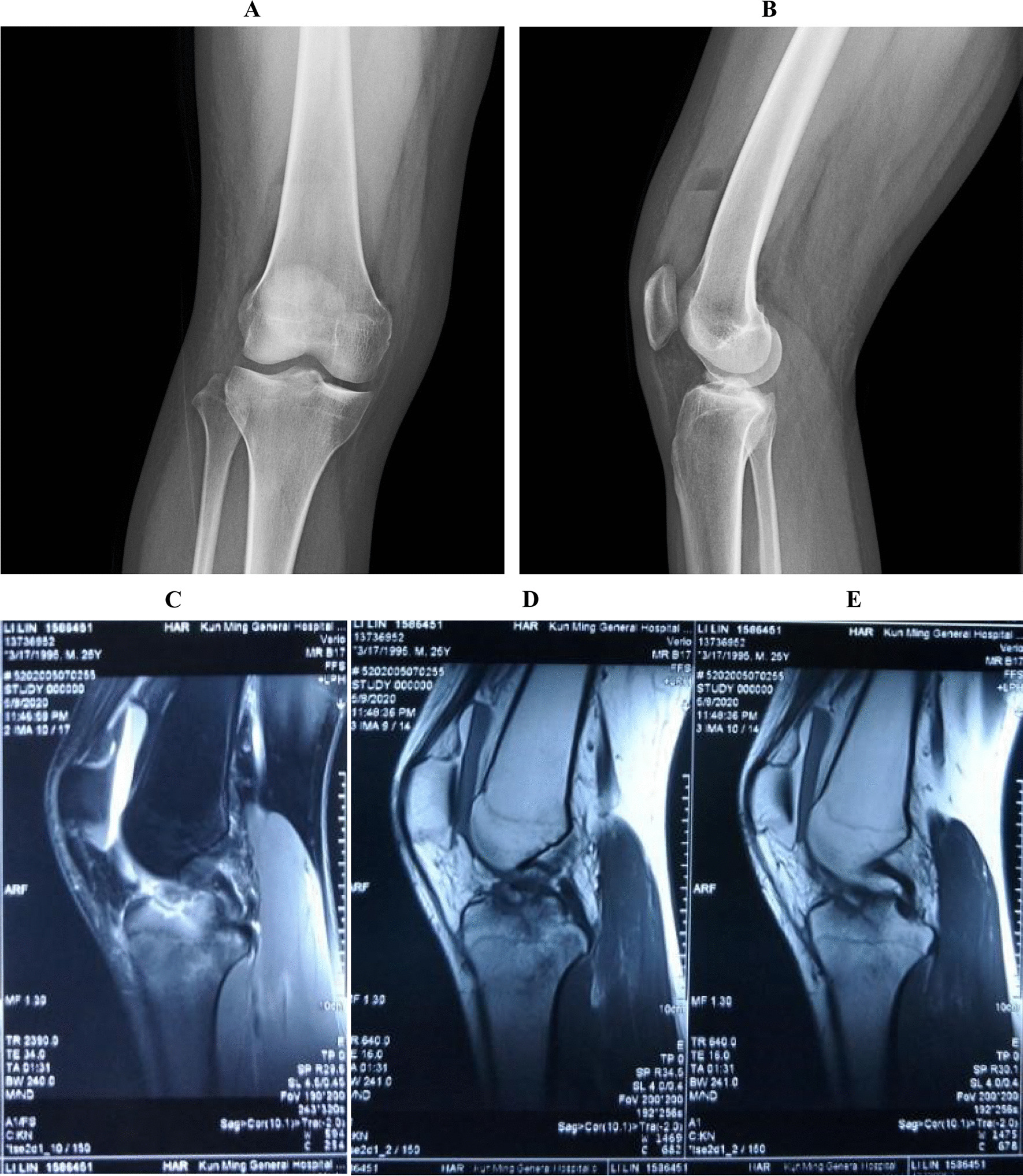
Preoperative detection results of cases. **A**, **B** X-ray detection of preoperative intercondylar ridge fracture in patients; **C**–**E** magnetic resonance imaging detection of intercondylar crest fracture before operation

Preoperative X-ray and magnetic resonance imaging examinations in the patient revealed the location, shape, and other joint injuries of the intercondylar crest fracture (Fig. 1).

**Fig. 2:**
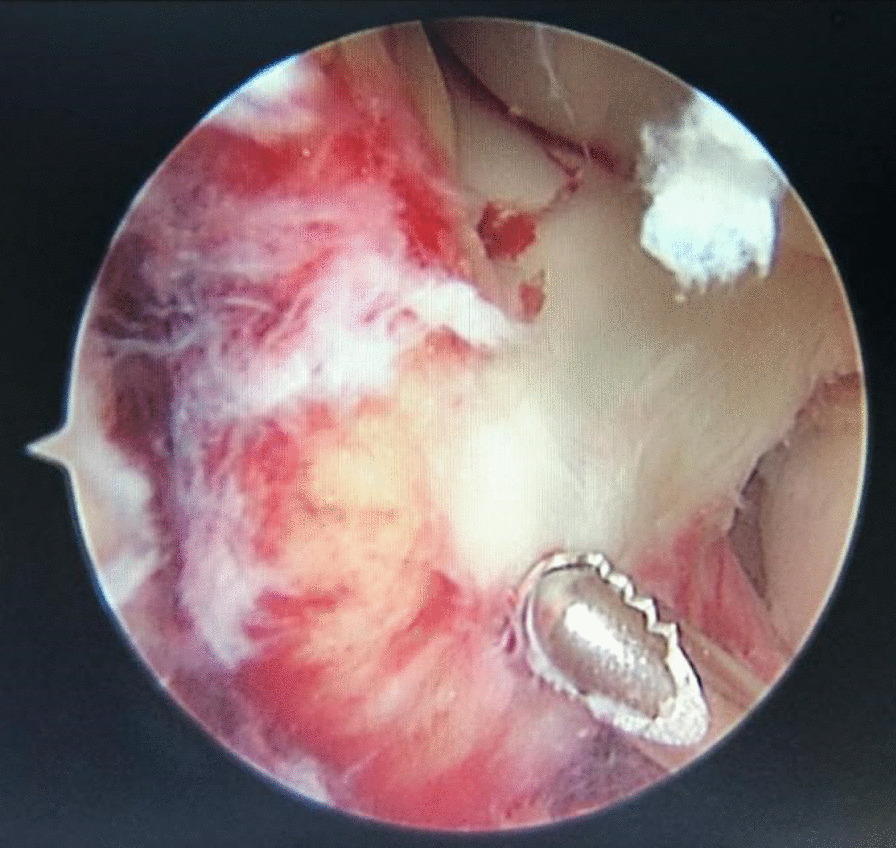
Cleaning of damaged joint

All patients were under general anesthesia. The patient was placed in the supine position, and bleeding was controlled with a tourniquet in the middle of the thigh to improve visualization. Before the operation, a thorough physical examination for the involved knee was performed. The tourniquet was inflated with a rubber elastic band to 100 mm Hg above the systolic blood pressure of the patient after exsanguination. Standard anteromedial and anterolateral approaches were established to remove cartilage debris from the articular cavity by thorough lavage. Routine diagnostic arthroscopies were performed using a standard 30 arthroscope to assess the associated injury. The hyperplastic synovial membrane was thoroughly cleaned to further expose the entire intercondylar fossa (Fig. 2).

**Fig. 3:**
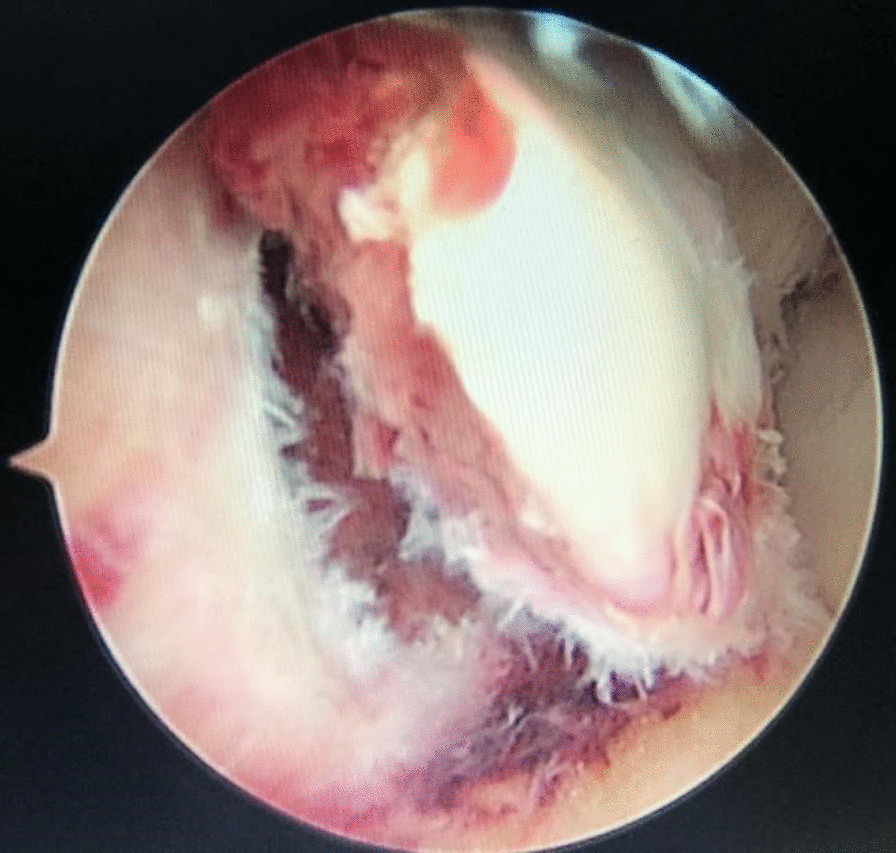
The location and characteristics of the lesion and the edge of the fracture block

Under arthroscopy, the anterior synovial membrane was cleaned, transverse ligament was fixed to one side and the anterior edge of the bulge fragment was exposed by turning over 30° and observing the forwardmost position of the tibial side. The medial and lateral crests of the original intercondylar eminence were gradually separated and pried out to completely expose the fracture bed (Fig. 3).

**Fig. 4:**
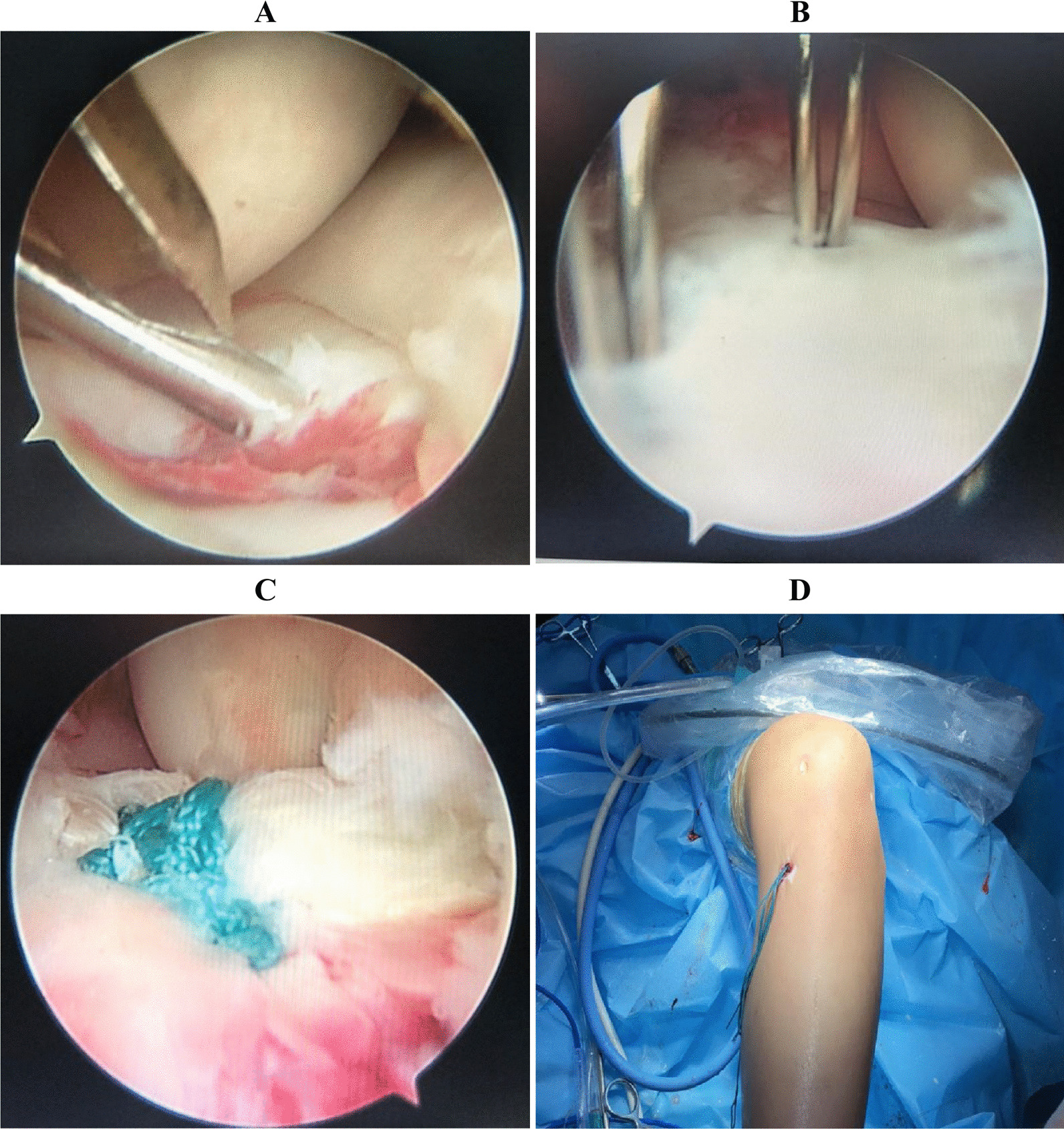
Surgical procedure. **A**, **B** The double-folded steel wire head through the 2.0 Kirschner opening hole; **C**, **D** the three double lines were completely led out

A 1.5-mm K-wire was used to temporarily fix the tibial eminence in its bed. The ACL tibial C-guide was applied through the anteromedial portal and its tip lying on 10 o’clock of the footprint of the fracture, and a 2-mm K-wire was used to drill a tibial tunnel directed from the proximal anteromedial tibial to the tip of the ACL guide. Another 2 tibial tunnels were drilled on 2 o’clock and 6 o’clock of the footprint of the fracture using same method. A No.5 ETHIBOND suture was through the ACL closed to the surface of the tibial eminence at 2 o’clock of the footprint of the fracture using a suture hook through the portal. A second b-junction suture No. 5 was threaded through the ACL at 6 o' clock, and the last b-junction suture No. 5 was threaded through the ACL at 10 o' clock, before which a respective navel ring was made and their limbs were individually pulled out of the respective tibial tunnels in the same manner as the first suture (Fig. 4A, B). Finally, the extremities of the three sutures are secured together on the anterior medial aspect of the proximal tibia. A 1.5-mm K-wire was removed, and arthroscopic verification of reduction of the intercondylar eminence and tension of the ACL was performed (Fig. 4C, D).

## Results

**Fig. 5:**
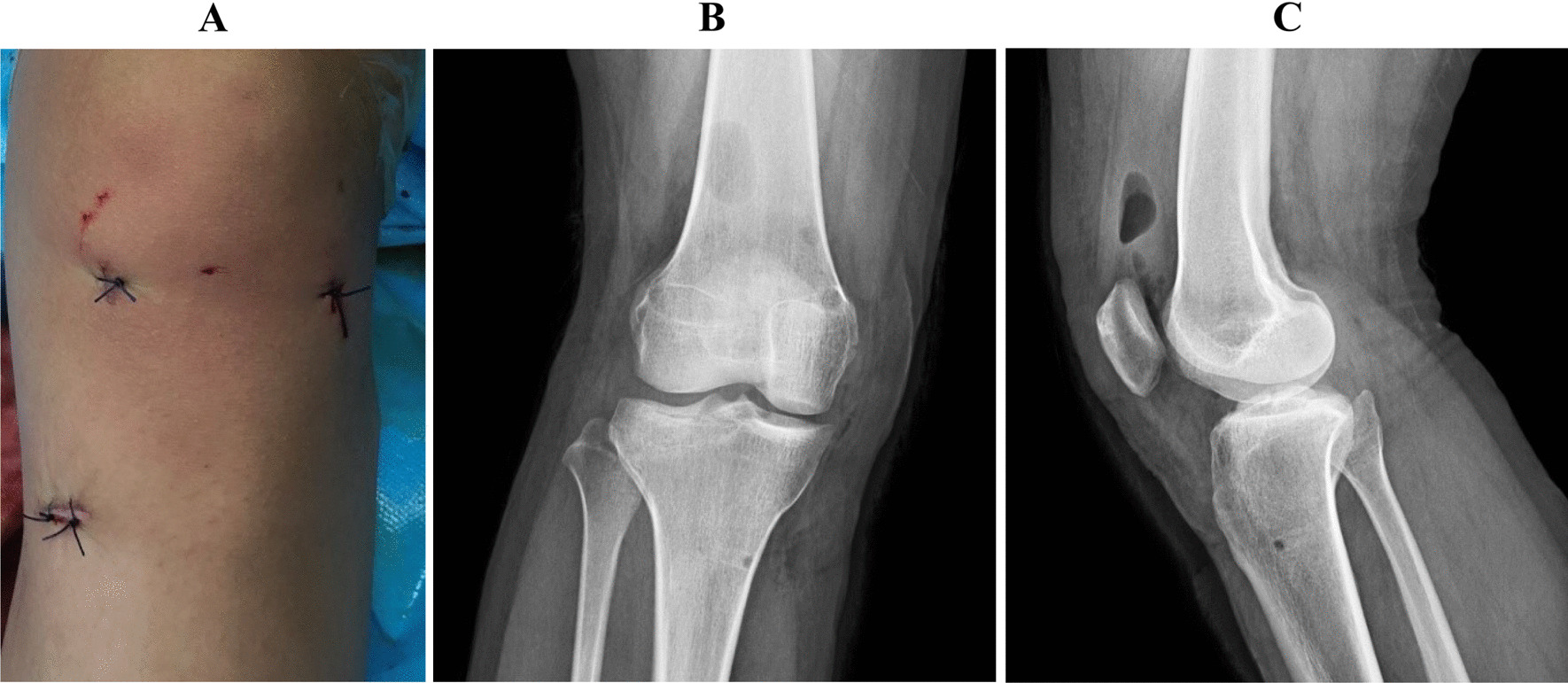
Recovery after operation. **A** The appearance of the incision after surgery was shown; **B**, **C** the results of radiographs were reviewed postoperatively

After surgical treatment, all patients followed standard postoperative protocols, including: (1) the knee joint was fixed in the static ACL knee joint bracket (0°) for 3 weeks, and the equal length quadriceps muscle was strengthened and straight leg training was carried out without weight bearing; (2) the hinged knee joint bracket was gradually flexed, and the knee joint was actively bent to 120° in 6 weeks; (3) partial weight bearing began at 6 weeks postoperatively; (4) gradually regained ambulation began at 2 months postoperatively, and full weight bearing was achieved at 3 months postoperatively. After the operation, the patients’ activities were obviously improved, and their fractures healed well (Fig. 5).

## Discussion

ACL avulsion is common in underdeveloped areas due to delayed diagnosis and inadequate repair facilities. Delay of operation is an important risk factor for articular fibrosis after surgery for tibial eminence fractures [18]. In a systematic review by Gans et al., there is insufficient evidence to conclude the superiority of open versus arthroscopic fixation or screw versus suture fixation techniques [19]. Although open reduction and internal fixation do not increase the risk of joint fibrosis and require less surgery, arthroscopic minimally invasive, almost scar-free surgery may be more attractive for adolescent patients, especially young girls.

At present, a variety of arthroscopic techniques have been reported for the fixation of tibial eminence fractures, for example, K-wires, cannulated screws, meniscal arrowheads, pullout sutures, anchor sutures, metal sutures, transosseous sutures, intra-articular buttons [1]. Arthroscopically assisted reduction and screw or suture fixation were the most commonly used and reliable surgical techniques reported for avulsion fractures of tibial intercondylar spinous dislocation. Callanan et al. compared clinical outcomes between suture and screw fixation for tibial avulsion fractures and found no difference in clinical or radiographic outcomes [20]. However, biomechanical studies have shown that compared with cannulated screws, suture fixation has higher peak failure and pullout strengths and is more stable after repeated cycling [21–23]. Screw fixation is generally suitable for large bone masses, but not suitable for comminuted fractures and small bone masses. In the process of operation, bone masses may be chopped up, resulting in internal fixation failure, and postoperative complications such as nail withdrawal may occur. Through the reduction of suture tension, suture fixation can obtain good fixation effect in all kinds of fracture morphology, has a wide range of applications, and will not appear in the operation of avulsion fracture fragment tear [24]. Another advantage of suture fixation is its relatively low cost, which allows the use of multiple sutures with minimal trauma, good reduction, firm fixation, and satisfactory clinical results with flexible suture technology.

There are some studies reported various suture fixation methods for tibial eminence fractures [12, 24–29]. These suture fixation methods use two or more sutures with one or more tibial tunnels or without tibial tunnel. Suture fixation with threaded screws has been reported with good clinical results [30]. Since the use of rivets with thread can penetrate deep into the bone and provide a good holding force, the high-strength suture can ensure that the bone is firmly fixed. The absorbable rivets are biodegradable and do not require a second operation to remove them, which reduce the patient’s psychological discomfort caused by the retention of implants and also eliminate some unnecessary psychological burdens, which meet the needs of patients for treatment. A study compared absorbable and non-absorbable suture fixation results in the treatment of tibial eminence fractures in children and adolescents and demonstrated similar clinical results in two groups [31]. In another clinical study, Delcogliano et al. compared PDS I 2.0 sutures to non-resorbable No. 2 ETHIBOND sutures for the treatment of adult tibial eminence fractures, and both groups had good clinical and functional outcomes [32]. But Schneppendahl et al. compared PDS II, Vicryl, and fiber wire for the fixation of tibial eminence fractures in children; in conclusion, they recommend Vicryl as a possible alternative to non-absorbable sutures but denied the same competence for PDS II [33].

Previous studies described various tibial tunnels for the suture pulled out for fixation. Elsaid et al. described the single tunnel pullout suture technique with internal buckle, and the authors believed that this technique was effective, economical, and short in operation time, providing a less invasive option for patients with immature bones [25]. However, in some previous studies, double tibial tunnels have been mostly used [27].

In this study, we used the improved three-tunnel pullout suture technique and considered that three-point fixation provided better proper reduction and stable fixation than two or one tunnel. This technique is applicable to all types of avulsion fractures of the tibial intercondylar eminence. Our modified technique is more amenable to reduction and stable fixation through a three-point fixation without implants, without the need for secondary surgery for removal, without complications associated with loosening of the intra-articular implant, and at a relatively low treatment cost, which can be used in patients with comminuted fractures. However, there are some limitations in our technology, such as the need for experienced arthroscopy, and the suture should penetrate the raised fragments and ACL.
